# Identification of Reliable Sulcal Patterns of the Human Rolandic Region

**DOI:** 10.3389/fnhum.2016.00410

**Published:** 2016-08-17

**Authors:** Charles Mellerio, Marie-Noël Lapointe, Pauline Roca, Sylvain Charron, Laurence Legrand, Jean-François Meder, Catherine Oppenheim, Arnaud Cachia

**Affiliations:** ^1^Department of Neuroradiology, Centre Hospitalier Sainte-AnneParis, France; ^2^University Paris DescartesParis, France; ^3^Imaging Biomarkers of Brain Development and Disorders, INSERM, UMR 894Paris, France; ^4^Department of Radiology, Hôpital de l’Enfant-Jésus, CHU de QuébecVille de Québec, QC, Canada; ^5^Laboratory for the Psychology of Child Development and Education, CNRS, UMR 8240Paris, France; ^6^Institut Universitaire de FranceParis, France

**Keywords:** central sulcus, pre-central sulcus, post-central sulcus, magnetic resonance imaging, cortex, sulcal patterns

## Abstract

A major feature of the human cortex is its huge morphological variability. Although a comprehensive literature about the sulco-gyral pattern of the central region is available from post-mortem data, a reliable and reproducible characterization from *in vivo* data is still lacking. The aim of this study is to test the reliability of morphological criteria of the central region sulci used in post-mortem data, when applied to *in vivo* magnetic resonance imaging (MRI) data. Thirty right-handed healthy individuals were included in the study. Automated segmentation and three dimensional (3D) surface-based rendering were obtained from clinical 3D T1-weighted MRI. Two senior radiologists labeled the three sulci composing the central region (precentral [PreCS], central [CS] and postcentral [PostCS]) and analyzed their morphological variations using 47 standard criteria derived from Ono’s atlas based on post-mortem data. For each criterion, inter-rater concordance and comparison with the occurrence frequency provided in Ono’s atlas were estimated. Overall, the sulcal pattern criteria derived from MRI data were highly reproducible between the raters with a high mean inter-rater concordance in the three sulci (CS: *κ* = 0.92 in left hemisphere/*κ* = 0.91 in right hemisphere; PreCS: *κ* = 0.91/*κ* = 0.93; PostCS: *κ* = 0.84/0.79). Only a very limited number of sulcal criteria significantly differed between the *in vivo* and the post-mortem data (CS: 2 criteria in the left hemisphere/3 criteria in the right hemisphere; PreCS: 3 in the left and right hemispheres; PostCS: 3 in the left hemisphere and 5 in the right hemisphere). Our study provides a comprehensive description of qualitative sulcal patterns in the central region from *in vivo* clinical MRI with high agreement with previous post-mortem data. Such identification of reliable sulcal patterns of the central region visible with standard clinical MRI data paves the way for the detection of subtle variations of the central sulcation associated with variations of normal or pathological functioning.

## Introduction

Sulci and gyri provide a natural topographic partition of the cortical anatomy. A huge inter- and intra-individual variability in the morphology of the cortical gyri and sulci—including size, shape and spatial pattern—has been previously reported (Ono et al., 1990; Rademacher et al., 1993; Rajkowska and Goldman-Rakic, 1995; Bartley et al., 1997; Blanton et al., 2001). Such variability raises methodological issues for functional brain mapping (Hunton et al., 1996) as well as for lesion localization in neurosurgical planning (Signorelli et al., 2001; Quiñones-Hinojosa et al., 2003). Difficulties in disentangling normal from abnormal morphological variations affect the detection of subtle dysmorphology associated with neurological or psychiatric conditions, e.g., neurodevelopmental impairments, schizophrenia (Yücel et al., 2002; Nakamura et al., 2007a; Plaze et al., 2011, 2015; Cachia et al., 2015) or malformation of cortical development (Barkovich et al., 2012).

The central region, located between the precentral sulcus (PreCS) and the postcentral sulcus (PostCS) and centered on the central sulcus (CS), provides major anatomo-functional landmarks. For instance, the CS hand knob is a stable morphological landmark (Yousry et al., 1997) and limits the primary motor area in the precentral gyrus and the primary somatosensory area in the postcentral gyrus. Descriptions of the central region and its variations have been based on visual inspection of post-mortem brains (Broca, 1888; Campbell, 1905; Cunningham, 1905). More recently, the sulcal anatomy from 25 post-mortem brains provided standardized morphological criteria to define and identify each sulcus of the human brain (Ono et al., 1990). This atlas provided by Ono introduced 47 criteria to characterize the central region sulci (PreCS, CS and PostCS). It is unclear, however, whether these criteria from post-mortem data can be transposed to *in vivo* data obtained from clinical magnetic resonance imaging (MRI). To this end, we evaluated the inter-rater concordance of each Ono criterion estimated from three dimensional (3D) reconstructions of brain surface (Mangin et al., 2004) obtained from anatomical MRI from 30 control subjects and compared their occurrence frequency with that from post-mortem data in Ono’s seminal study (Ono et al., 1990). Only the standard Ono atlas was used in this study as it is the only atlas that provides quantitative description (i.e., distribution percentage) of the morphological sulcal pattern variations.

## Materials and Methods

### Subjects

Thirty healthy, right-handed individuals (14 men, 16 women; median age = 30 years, range = 22–50 years) were participants in the study. Imaging was performed with the informed written consent of the participants and the approval of the ethics committee Ile-de-France III.

### MRI Acquisition

In all the subjects, images were acquired on a 1.5 T Signa MR scanner (Signa 1.5T, General Electrics Healthcare, Milwaukee, WI, USA) using an inversion recovery 3D T1-weighted fast-spoiled gradient recalled acquisition (repetition time/echo time/flip angle: 10/2 ms/15°, 1.2 mm slice thickness, no gap, in-plane resolution: 0.93 × 0.93 mm, acquisition time: 6 min 14 s).

### Image Analysis

In order to generate 3D images of the sulci, the raw MRI data were subjected to automatized segmentation (Mangin et al., 2004) and 3D surface-based rendering using BrainVisa software^1^ using standard parameters. Briefly, an automated pre-processing step skull-stripped T1-weighted MRI, segmented the brain tissues (cerebrospinal fluid, gray matter, and white matter), separated the two hemispheres and reconstructed the 3D surfaces corresponding to the gray–white matter and gray matter–cerebrospinal fluid interface. The cortical folds were then automatically segmented throughout the cortex from the skeleton of the gray matter–cerebrospinal fluid mask, with the cortical folds corresponding to the crevasse bottoms of the “landscape”, the altitude of which is defined by the intensity on MRI. This definition provides a stable and robust sulcal surface definition that is not affected by variations of the gray–white matter contrast. The cortical folds were then converted to a graph-based representation of the cortex containing information relating to shape (area, depth and length) and spatial organization (position and orientation). No spatial normalization was applied to the MRI data to overcome potential bias due to the sulcus shape deformations induced by the warping process.

### Sulcal Characterization

The three sulci of the central region (preCS, CS and postCS) were manually labeled and analyzed independently by two senior radiologists (CM and M-NL) based on the presence or absence of the 47 morphological criteria used in Ono’s atlas (Ono et al., 1990). Only anatomical landmark derived from standard clinical MR images were used to identify the sulci. Table 1 provides a description of the 16 criteria defining the CS, Table 2 the description of the 18 criteria defining the PreCS and Table 3 the description of the 13 criteria defining the PostCS.

**Table 1:** **Description of the 16 criteria defining the pattern of the CS**.

Region/pattern	Criteria	Description
Whole sulcus	Continuous or discontinuous	Interruption (discontinuity) is caused by a transitional convolution
Inferior end	Extension into the SF	Connection between CS and SF
	Anterior direction	Compared to the general axis of the central sulcus
	Posterior direction
	Straight shape	General shape of the inferior end of the sulcus
	“Y” shape
	“T” shape
Superior end	Extension into the medial surface
	Straight shape	General shape of the superior end of the sulcus
	“Y” shape
	“T” shape
Side branches	Over precentral gyrus	Small sulci having the same depth as CS, cutting anteriorly
	Over postcentral gyrus	Small sulci having the same depth as CS, cutting posteriorly
Connections	With precentral sulcus	Connections result from the union of two sulci that run in the same direction and at the same level
	With postcentral sulcus	
	With small free sulcus in precentral gyrus

*SF, Sylvian fissure; CS, central sulcus*.

**Table 2:** **Description of the 18 criteria defining the pattern of the preCS**.

Region/Pattern	Criteria	Description
Whole sulcus	Number of segments	Interruption between segments are caused by a transitional convolution
Marginal PreCS	Present or absent	Horizontally oriented sulcus, over the lateral surface and above the superior side of the preCS
Medial PC sulcus	Present or absent	Notches the superior margin of the hemisphere above the superior preCS.
		Of note, two medial precentral sulci can coexist in the same hemisphere (on each side of the preCS axis)
Superior segment	Arcuate termination with Y-shaped end	According to the sulcus curvature (arcuate or T-shaped) and the end shape (straight or Y-shaped)
	T-shaped side anastomosis with Y-shaped end	
Inferior segment	Arcuate form	According to the general shape of the sulcus
	Ramified form
	Bayonet form
	Y-shaped end
Inferior end	Extension into SF
	Straight shape
	Y-shape
Connections	With central sulcus	Connections result from the union of two sulci that run in the same direction and at the same level
	With superior frontal sulcus	
	With intermediate frontal sulcus
	With inferior frontal sulcus

*SF, Sylvian fissure; preCS, precentral sulcus*.

**Table 3:** **Description of the 13 criteria defining the pattern of the postCS**.

Region/Pattern	Criteria	Description
Whole sulcus	Number of segments	Interruption between segments are caused by a transitional convolution
Inferior end	Extension into SF	Connection between postCS and SF
Double parallel pattern	With intraparietal sulcus	Orientation of the postCS parallel to the intraparietal sulcus
	With posterior subcentral sulcus	Orientation of the postCS parallel to the posterior subcentral sulcus
Superior end	Y-shape	According to the general shape of the sulcus
	Straight
	Extension to the medial surface
Side branches	Over postcentral gyrus	Small sulci having the same depth as postCS, cutting anteriorly or posteriorly
	Over superior parietal lobule
	Over inferior parietal lobule
Connections	With central sulcus	Connections result from the union of two sulci that run in the same direction and at the same level
	With intraparietal sulcus
	With superior temporal sulcus

*SF, Sylvian fissure; postCS, postcentral sulcus*.

### Statistical Analysis

For each of the 47 Ono’s criteria in the left and right hemispheres, we estimated: (1) the inter-rater concordance (kappa index); and (2) the difference with the data obtained by Ono from post-mortem data (Chi square tests).

## Results

### Inter-Rater Concordance for *In Vivo* Data

The mean inter-rater concordance (kappa index) was calculated for all sulcal pattern criteria.

For the CS, mean inter-rater concordance was excellent in both left (*κ* = 0.92, range: 0.77–1.0) and right (*κ* = 0.91, range: 0.77–1.0) hemisphere (Table 4). The concordance was good only for the number of sulcal branches over the precentral gyrus (*κ* = 0.77).

**Table 4:** **Mean occurrence frequency and inter-rater concordance of each criterion of the CS in the left and right hemispheres**.

Criteria	Variations	Left hemisphere		Right hemisphere	
		Mean (%)	κ	Mean (%)	κ
Interruptions	Continuous	97	**1**	97	**1**
Inferior end	Extension into the SF	13	**0.96**	17	**0.92**
	Anterior direction	40	**0.86**	53	0.79
	Posterior direction	60	**0.86**	47	0.79
	Straight shape	100	**0.82**	90	**0.94**
	“Y” shape	0	**1**	10	**0.95**
	“T” shape	0	**0.91**	0	**1**
Superior end	Extension into the medial surface	57	**0.80**	50	**0.80**
	Straight shape	100	**1**	93	**0.94**
	“Y” shape	0	**1**	3	**0.95**
	“T” shape	0	**1**	3	**0.95**
Side branches	Over precentral gyrus
	0	67		67	
	1	27		20
	2	3	0.77	10	0.77
	3	3		3
	4	0		0
	Over postcentral gyrus
	0	85		83
	1	13		17
	2	2	**0.88**	0	**0.84**
	3	0		0
	4	0		0
Connections	With precentral sulcus	27	**0.92**	10	**0.95**
	With postcentral sulcus	0	**1**	0	**1**
	With small free sulcus in preC gyrus	0	**0.95**	7	**0.95**

*Sulcal criteria with high inter-rater concordance (κ > 0.8) are shown in bold font. Measures with a significant difference between in vivo and ex vivo data values (p-value < 0.05) are underlined. preC, precentral*.

For the PreCS, mean inter-rater concordance was also excellent in both left (*κ* = 0.91, range: 0.76–1.0) and right (*κ* = 0.93, range: 0.61–1.0) hemisphere (Table 5). It was good only for the left medial and superior segment variation and the right inferior end variation (*κ* < 0.8).

**Table 5:** **Mean occurrence frequency and inter-rater concordance of each sulcal criterion of the preCS in the left and right hemispheres**.

Criteria	Variations	Left hemisphere		Right hemisphere	
		Mean (%)	κ	Mean (%)	κ
Number of segments	2	60	**0.95**	70	**1**
	3	37	**0.94**	30	**1**
	4	3	**1**	0	**1**
Marginal PreCS		30	**0.89**	37	**0.90**
Medial PreCS	0	13	**0.83**	23	**0.90**
	1	60	0.77	53	**0.86**
	2	27	**0.89**	23	**0.98**
Superior segment	Arcuate termination	43	**0.82**	33	**0.95**
	With Y-shaped end	10	**0.93**	10	**1**
	T-shaped side anastomosis	33	0.77	47	**0.91**
	With Y-shaped end	13	0.76	10	**0.93**
Inferior segment	Arcuate form	33	**0.86**	17	**0.94**
	Ramified form	33	**0.90**	40	**0.95**
	Bayonet form	10	**0.88**	27	**1**
	Y-shaped end	23	**0.94**	17	**1**
Inferior end	Extension into SF	60	**0.90**	50	**1**
	Straight shape	97	**1**	90	0.61
	Y-shape	3	**1**	10	0.61
Connections	With CS	27	**0.91**	10	**1**
	With superior FS	80	**1**	73	**0.91**
	With intermediate FS	23	**1**	27	**0.91**
	With inferior FS	77	**1**	57	**1**

*Sulcal criteria with high inter-rater concordance (κ > 0.8) are shown in bold font. Measures that significantly differ between in vivo and ex vivo data values (p-value < 0.05) are underlined. PreCS, precentral sulcus; CS, central sulcus, SF, sylvian fissure; FS, frontal sulcus*.

Finally, for the PostCS, the mean inter-rater concordance was globally excellent with *κ* = 0.84 (range: 0.59–1.0) for the left hemisphere and 0.79 (range: 0.60–1.0) for the right hemisphere (Table 6), even though the concordant was good only for several criteria (left number of segments, superior end variation, side branches over postcentral gyrus and right number of segments, inferior and superior end variation, and number of side branches over postcentral gyrus and inferior parietal lobule).

**Table 6:** **Mean occurrence frequency and inter-rater concordance of each criterion of the postCS in the left and right hemispheres**.

Criteria	Variations	Left hemisphere		Right hemisphere	
		Mean (%)	κ	Mean (%)	κ
Number of segments	Continuous	60	**0.92**	73	0.73
	Two	30	**0.84**	20	0.79
	Three	10	0.78	7	0.65
Inferior end	Extension into SF	40	**0.92**	37	0.71
Double parallel pattern	With intraparietal sulcus	27	0.59	33	**0.92**
	With posterior subcentral sulcus	3	**1**	7	**1**
Superior end	Y-shape	43	0.79	27	0.64
	Straight	57	0.79	73	0.64
	Extension to the medial surface	27	0.65	30	0.73
Side branches	Over postcentral gyrus
	0	17	0.72	33	0.74
	1	47		50
	2	20		10
	3	13		7
	4	3		0
	5	0		0
	Over superior parietal lobule
	0	17		18
	1	12	**0.93**	12	**0.86**
	2	3		0
	Over inferior parietal lobule
	0	77		87	
	1	23	**0.90**	13	0.60
	2	0		0
Connections	With CS	0	**1**	0	**1**
	With intraparietal sulcus	80	**0.88**	77	**0.83**
	With superior temporal sulcus	0	**1**	17	**1**

*Sulcal criteria with high inter-rater concordance (κ > 0.8) are shown in bold font. Measures with a significant difference between in vivo and ex vivo data values (p-value < 0.05) are underlined. PostC, postcentral; CS, central sulcus; SF, Sylvian fissure*.

### Comparison of *In Vivo* and Post-Mortem Data

Overall, 80% of the criteria in the *in vivo* analysis did not differ from the analysis based on Ono’s data regarding their frequency of occurrence. Only a limited number of sulcal criteria significantly differed (*p* < 0.05) between the *in vivo* and the post-mortem data: there were 3 (18%) (resp. 2 [12%]) in the left (resp. right) CS (Table 4), 3 (13%) (resp. 3 [13%]) in the left (resp. right) PreCS (Table 5) and 3 (20%) (resp. 5 [33%]) in the left (resp. right) PostCS (Table 6).

The criteria that differed between Ono’s post-mortem data and *in vivo* MRI data were:

– for the CS: inferior end straight shape, inferior end T-shape and the number of connections with small free sulcus in the left precentral gyrus;
– for the preCS: extension to the left Sylvian fissure (SF), number of connections with left CS, right superior segment anastomosis, inferior end arcuate form and connections with right frontal sulcus (FS);
– for the post CS: left and right superior end variations, right inferior end variation and right double parallel pattern.

**Figure 1:**
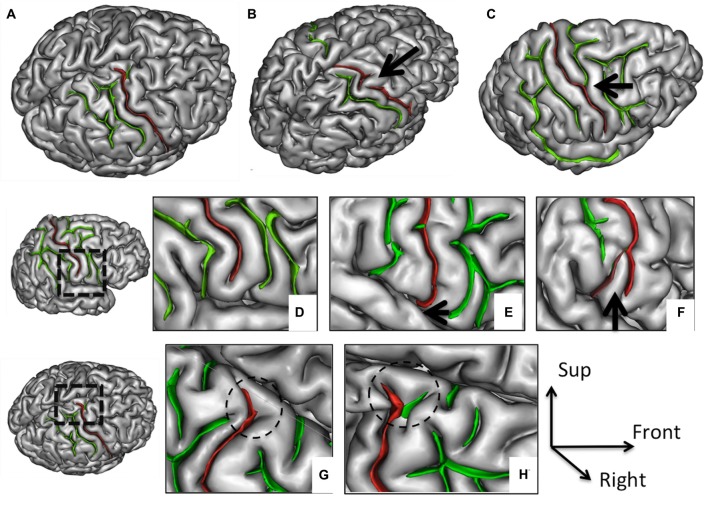
**Morphological features of the central sulcus (CS) with an excellent inter-rater concordance (κ > 0.80) and with values (frequency occurrence) that did not differ from Ono’s post-mortem values.** The CS (in red) is represented on a three dimensional (3D) mesh-based reconstruction of the cortex surface. Continuous **(A)** or interrupted (**B**, arrow) CS. Connection with the precentral sulcus (PreCS; **C**, arrow). Inferior end without **(D)** or with extension to the sylvian fissure (SF; **E**, arrow). Inferior end “Y” shape **(F)**. Superior end: “T” shape **(G)** or “Y” shape **(H)**.

**Figure 2:**
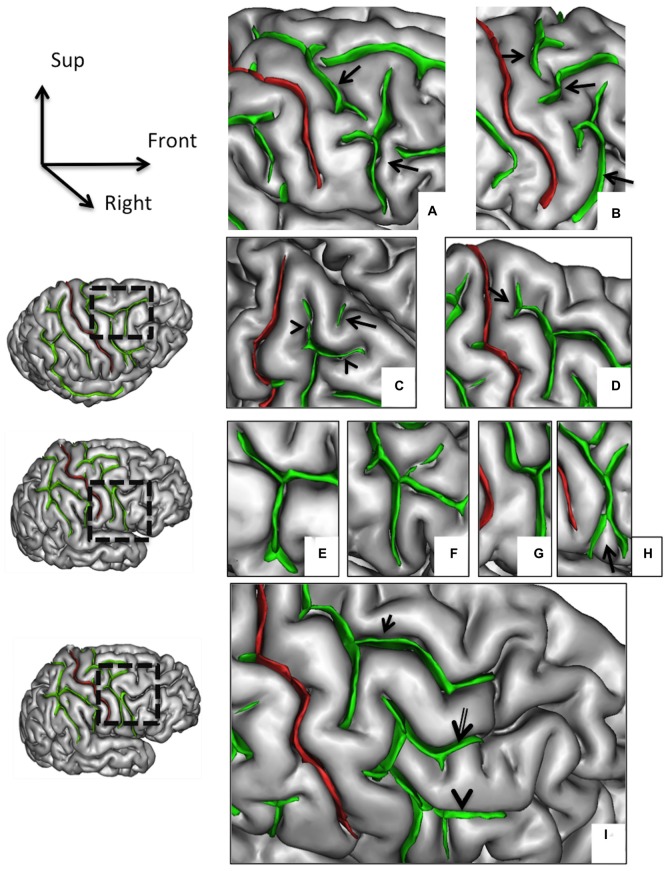
**Morphological features of the precentral sulcus (PreCS) with an excellent inter-rater concordance (κ > 0.80) and with values (frequency occurrence) that do not differ from Ono’s post-mortem values.** The PreCS (in green) represented on a 3D mesh-based reconstruction of the cortex surface. PreCS with two **(A)** or three **(B)** segments (arrows). PreCS superior end patterns **(C)** with marginal PreCS (arrow heads) and medial PreCS (arrow). PreCS superior segment shape with arcuate termination with Y-shaped end (**D**, arrow). Pre-CS inferior segment patterns with arcuate form **(E)**, ramified form **(F)**, bayonet form **(G)** and Y-shaped end (**H**, arrow). PreCS connections **(I)** with superior frontal sulcus (arrow), intermediate frontal sulcus (double arrow) or inferior frontal sulcus (arrow head).

**Figure 3:**
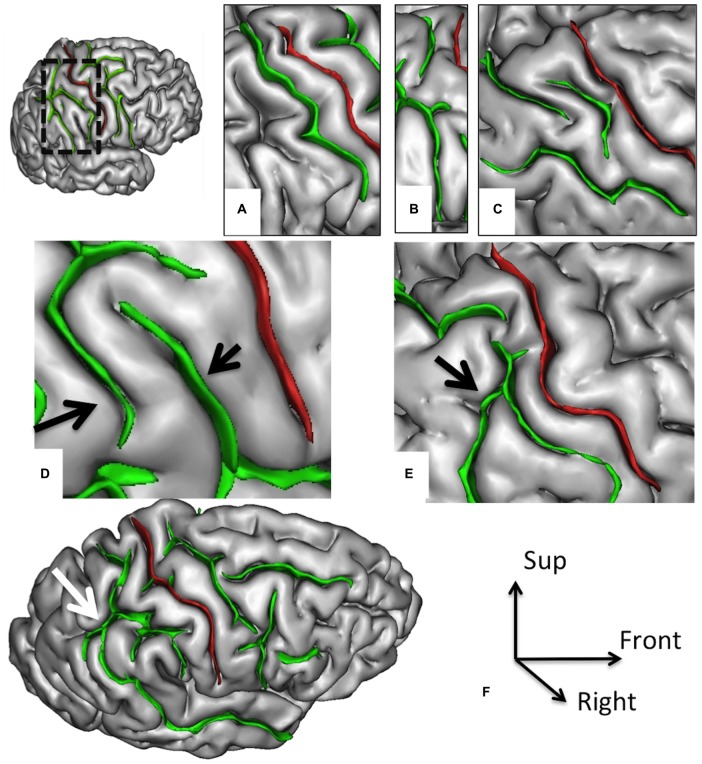
**Morphological features of the postcentral sulcus (PostCS) with an excellent inter-rater concordance (κ > 0.80) and with values (frequency occurrence) that do not differ from Ono’s post-mortem values.** The PostCS (in green) represented on a 3D mesh-based reconstruction of the cortex surface. PostCS with one **(A)**, two **(B)** or three **(C)** segments. PostCS double parallel pattern (**D**, arrows). PostCS connections with intraparietal sulcus (**E**, arrow) or with superior temporal sulcus (**F**, arrow).

Details of features that showed excellent inter-rater concordance (κ > 0.80; Landis and Koch, 1977) and that did not differ from Ono’s post-mortem values are represented in Figure 1 for the CS, Figure 2 for the Pre-CS and Figure 3 for the Post-CS.

## Discussion

The present MRI study on the sulcal variability in the central region confirms the sulcal pattern distributions reported in Ono’s atlas and its applicability to MRI studies. It provides two main findings: (1) most of the 47 sulcal criteria characterizing the sulcal morphology of the central region, initially developed by Ono for post-mortem data (Ono et al., 1990), can be used for *in vivo* MRI data; (2) analysis of *in vivo* (MRI) and *ex vivo* (post-mortem) data provide similar variations of the normal sulcal anatomy of the CS, preCS and postCS. The most noticeable difference between the analysis based on 3D MR images and the analysis based on post-mortem data concerns the number of side branches and sulcal connections, with more indentations in the post-mortem description than in the MRI analysis. Such differences likely result from a lower spatial resolution in MRI data compared to *ex vivo* data, and from a post-processing regularization step (spatial pruning in the sulcal skeleton of small folds) used to limit the effect of a minor segmentation error (Mangin et al., 1995).

### Relevance for Clinical Studies

By providing reference data on normal variations of MRI-derived sulcal features of the central area, this work has important clinical applications as it allows clinicians detecting unusual sulcal patterns. This may be useful, for instance, in patients referred for intractable epilepsy and suspected of having focal cortical dysplasia (FCD) in the central region with a normal MRI based on conventional analysis. Up to 50% of such dysplasia affect the central region (Mellerio et al., 2012). Sulcal abnormalities have been reported in FCD, such as abnormally deep sulci (Besson et al., 2008). Such features are difficult to be detected by the human eye and are likely underestimated due to the absence of standard criteria to disentangle normal from abnormal sulcal patterns. Based on the visual analysis of the 3D surface rendering MR images, one study describes a specific sulcal pattern associated with the presence of an FCD in the central region (Mellerio et al., 2015) even in patients with “normal” MRI. The present study could provide reference data for such an individual sulcal-based analysis. For instance, in a patient referred for intractable epilepsy and suspected of having cortical dysplasia in the central region, the presence of an unusual value for one or several morphological criteria could help to detect an underlying lesion, even in the case of negative MRI.

In addition to neurological conditions, the analysis of the central region sulcation may also be of interest in psychiatric disorders, notably in schizophrenia. Indeed, motor impairments have been reported in schizophrenia (Strube et al., 2014), but it is still not clear to what extent they can be attributed to the anatomy of the central region. Hence, neurological soft signs—i.e., observable defects in motor coordination, motor integration and sensory integration—have been reported at all stages of schizophrenia, including in first episode psychosis patients and in antipsychotic naïve patients (Bombin et al., 2005). A recent study of schizophrenia patients detected an association between the presence of neurological soft signs and the sulcal surface area in several cortical regions but not in the central region (Gay et al., 2013). It will be interesting to test whether some sulcal descriptors of the central region identified in the present study, which are more reliable than a simple measure of surface area, can pinpoint abnormal sulcal patterns of CS, preCS or postCS associated with neurological soft signs.

### Investigating the Long Term Effect of Sulcal Region Ontogenesis

The sulcal pattern results from early processes during fetal life that shape the cortex anatomy from a smooth lissencephalic structure to a highly convoluted surface (Mangin et al., 2010). Several genetic and environmental factors (Dehay et al., 1996; Molko et al., 2003; Rakic, 2004; Barkovich et al., 2012) contribute to the neurodevelopmental processes that influence the shape of the folded cerebral cortex, including structural connectivity through axonal tension forces (Van Essen, 1997; Hilgetag and Barbas, 2006). These mechanical constraints lead to a compact layout that optimizes the transmission of neuronal signals between brain regions (Klyachko and Stevens, 2003) and thus brain network functioning. This association between cortical folding and network functioning may explain why the sulcal pattern is an early marker of later functional development (Dubois et al., 2008).

The qualitative features of the sulcal pattern, like the 47 criteria of the central region investigated in the current study, are markers of early brain development (Welker et al., 1988). Indeed, as opposed to *quantitative* measures of the cortex anatomy—such as the Gyrification Index (Armstrong et al., 1995; White et al., 2010; Zilles et al., 2013) or the thickness, surface, and volume of the cortex (Gogtay et al., 2004; Giedd et al., 2009) that vary from childhood through early adulthood—the sulcal pattern is a stable feature of the brain anatomy not affected by brain maturation occurring after birth (Sun et al., 2012; Cachia et al., 2016). The analysis of the qualitative features of the sulcal pattern therefore raises new possibilities to investigate the long term effect of fetal life on symptom variability in neurological (Kim et al., 2008; Régis et al., 2011; Roca et al., 2015) and psychiatric (Yücel et al., 2002; Nakamura et al., 2007b; Cachia et al., 2008; Plaze et al., 2015) disorders and also on normal variability in cognitive efficiency in healthy subjects (Cachia et al., 2014; Borst et al., 2016).

The exact link between the folding pattern and functional competence is a complex issue (Welker et al., 1988; Zilles et al., 2013). However, strong correspondences between cortical folding features and functional activations have been found not only in primary areas but also in higher level areas, including the visual areas (Watson et al., 1993; Dumoulin et al., 2000), the paracentral sulcus (Grosbras et al., 1999), the PreCS (Sun et al., 2015), the frontal operculum (Amiez et al., 2016), the midcingulate cortex (Amiez et al., 2013), the dorsal premotor region (Amiez et al., 2006) and the fusiform gyrus (Weiner et al., 2014). The main difficulty limiting the investigation of such sulcal morphology/function correspondence is the variability of the folding pattern (Ono et al., 1990; Régis et al., 2005; Petrides et al., 2012). A critical issue is therefore the possible identification of reliable sulcal patterns from MRI data.

### Limitations

The results of this study are best understood in the context of some methodological issues. Firstly, only right-handed subjects were selected in this study in order to optimize the sample homogeneity and limit the reported handedness-related bias on the sulcal anatomy, notably in the central region (Amunts et al., 2000; Klöppel et al., 2010; Sun et al., 2012). Further studies, investigating sulcal patterns in left-handed as well as mixed-handed subjects are needed. Secondly, the so-called “plis de passage”–small gyri deeply buried in the main sulci (Gratiolet, 1854; Broca, 1888; Cunningham, 1892) associated with U-shaped white matter fibers—are important landmarks to characterize the sulcal patterns (Régis et al., 2005) but were not used in this study because their detection from structural MRI is difficult (Cachia et al., 2003). Future multimodal analyses, combining 3D reconstruction of the cortical surface from structural MRI with white-matter bundle tractography from diffusion MRI, should help to clarify the interactions between the sulcal patterns and the underlying white matter connectivity (Van Essen, 1997; Hilgetag and Barbas, 2006). In addition, it would be interesting to investigate the use of microstructural information (e.g., cortical myelination Glasser et al., 2014; Lutti et al., 2014) along with morphological features of the cortical surface for sulcal identification. Furthermore, comparison of *in vivo* and *ex vivo* data is a critical issue for translating the findings obtained from cadaver to living human brain. Hence, recent brain imaging approaches for “*in vivo* Brodmann mapping” allowing direct correlations between microstructure and function in living human brains (Geyer et al., 2011) would represent a major step forward for the brain mapping of human brain.

In conclusion, our study provides a description of sulcal patterns in the central region from *in vivo* clinical MRI which is in high agreement with previous *ex vivo* data. Such identification of reliable sulcal patterns of the central region visible with standard clinical MRI data paves the way for the detection of subtle variations of the central sulcation associated with variations of normal or pathological functioning.

## Author Contributions

CM, CO and AC contributed to the conception of the work. CM, CO, M-NL, LL, J-FM participated in the acquisition of the data. CM, AC, CO, PR, SC participated in the analysis and interpretation of the data. CM, CO and AC drafted the work. M-NL, PR, SC, LL, J-FM revised the work critically for important intellectual content. All authors approved the final version of the article and agreed to be accountable for all aspects of the work in ensuring that questions related to the accuracy or integrity of any part of the work are appropriately investigated and resolved.

## Conflict of Interest Statement

The authors declare that the research was conducted in the absence of any commercial or financial relationships that could be construed as a potential conflict of interest.

## Footnotes

^1^www.brainvisa.info
